# Mechanical Performance of Rotationally Molded Multilayer mLDPE/Banana-Fiber Composites

**DOI:** 10.3390/ma16206749

**Published:** 2023-10-18

**Authors:** Jake Kelly-Walley, Zaida Ortega, Mark McCourt, Bronagh Millar, Luis Suárez, Peter Martin

**Affiliations:** 1 Matrix Polymers, Unit 2, Compass Industrial Park, Spindus Road, Speke, Liverpool L24 1YA, UK; 2 Polymer Processing Research Centre, School of Mechanical and Aerospace Engineering, Queen’s University Belfast, Ashby Building, Stranmillis Road, Belfast BT9 5AH, Northern Ireland, UK; m.mccourt@qub.ac.uk (M.M.); b.millar@qub.ac.uk (B.M.); p.j.martin@qub.ac.uk (P.M.); 3 Departamento de Ingeniería de Procesos, Universidad de Las Palmas de Gran Canaria, Edificio de Ingenierías, Campus Universitario de Tafira Baja, 35017 Las Palmas de Gran Canaria, Spain; 4 Departamento de Ingeniería Mecánica, Universidad de Las Palmas de Gran Canaria, Edificio de Ingenierías, Campus Universitario de Tafira Baja, 35017 Las Palmas de Gran Canaria, Spain; luis.suarez@ulpgc.es

**Keywords:** rotomolding, composites, natural fibers, banana fibers, agro-industrial residues, sustainability, polyethylene

## Abstract

The incorporation of materials different from the polymer within the rotational molding process usually results in lowered mechanical properties, where impact strength is of particular concern. In order to overcome this issue, multilayer structures of virgin polyethylene (PE) and banana fiber composites were prepared to determine the impact of the different layers on the performance of the final part. Cycle time has been studied to identify the influence of the addition of fibers in the process. The tensile, flexural and impact properties have been analyzed, finding improvements in Young’s modulus of up to 13%, although at the expense of significant decreases in impact strength. A reduction in the fiber size due to the pulverization process was observed, which affected the rheological and mechanical behavior of the composite. The beneficial effects of working in multiple layers have been demonstrated in this work, where composites with up to 5% of banana fiber have been produced in two-layer structures. Finally, the need to add neat polyethylene in the external layer is also highlighted as a way to counteract the reductions in mechanical properties, particularly for flexural elastic modulus and tensile strength, and this also helps with the drop in impact behavior to a lower extent.

## 1. Introduction

Rotational molding (RM) is a polymer process method used to manufacture large, hollow, stress-free products [1,2]. The process can be referred to as rotational molding, Roto-molding or Roto [2]. Characteristically, the process has long cycle times, elevated temperatures and a lack of shear. However, there are many advantages to the process, such as benefits in short production runs, minimal waste, relatively inexpensive tooling and even wall thickness [2,3].

The rotational molding process is dominated by polyethylene (PE) in powder form; an estimated 85–90% of the material used in rotational molding is expected to be PE [2,4]. This is due to the thermal and rheological characteristics of the material and its durable properties lending themselves to both the process and applications.

**Figure 1:**
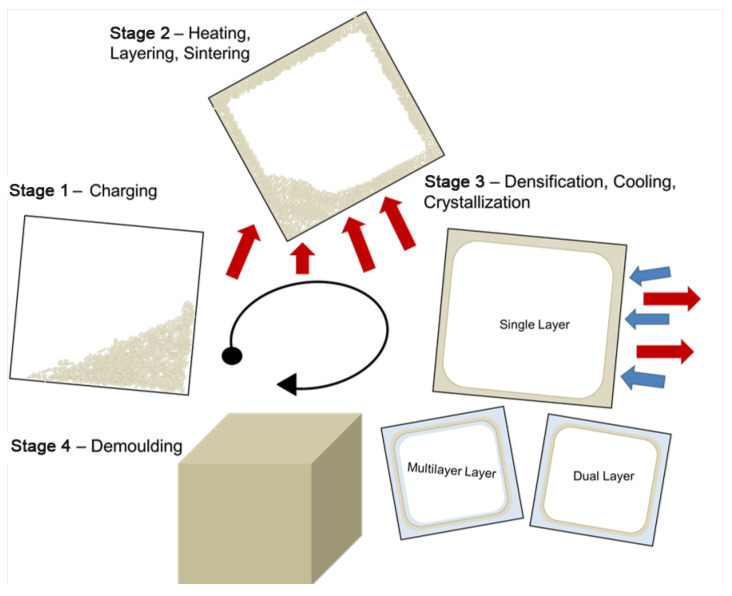
Schematic of the rotational molding process from charging the tool to de-molding the final molded part.

The process has different stages, which can be simplified into four fundamental ones [5,6]. Stage 1 is charging, or loading the tool with the material required to manufacture the product. Sequentially, stage 2 results in the closed tool entering an oven, usually rotating biaxially. During this time, the internal temperature of the tool rises, causing the polymer to absorb heat and begin to stick to the tool. The material will start to sinter and coalescence, forming a homogenous melt structure, allowing any trapped bubbles or air voids to escape from the bulk [4,5,7]. After heating, the tool is removed from the oven, starting Stage 3. Here, the internal air temperature decreases due to a drop in ambient temperature; the polymer will crystallize and form a solid part before the final stage [2]. In Stage 4, the part is de-molded and final finishing is applied if needed. Figure 1 presents a brief outline of the process. This technique can also be used to manufacture multilayer parts, in which case Stage 1 and 2 would be repeated [3].

The literature on rotational molding can be described as limited compared to other process methods. Despite the partial lack of research on rotational molding, in the past 10 years, there has been a growing interest in natural fibers within the rotational molding process, with over 35 publications in this area since 2020, which appears to continue growing year on year.

The increase in the number of studies is due to the interest in ‘greener’ or potentially more sustainable solutions to polymer composites and polymer reinforcement, as well as in widening the range of materials and properties. Given today’s environmental issues and the use (and sometimes abuse) of the term ‘sustainability’, it is always beneficial to understand why natural fibers are sustainable [8]. Firstly, it is reported that natural fibers use less than 18% CO_2_ in production compared to their synthetic counterparts [9]. In addition to this, the benefits and considerations needed to ensure these materials are sustainable are shown in Table 1.

In the case of this study, the banana fibers are waste products from the commercial growth of bananas for consumption. By adopting these fibers in composites, a reduction in natural waste and in polymer consumption is achieved, also potentially improving mechanical behavior due to the reinforcing character of such fibers. The interest in using fibers within the rotational molding process is clear in the literature, as a way not only to increase the range of materials and properties achieved, but also to reduce the amount of plastic used within a particular part. Further benefits include cost reduction and improved functionality, i.e., insulating thermal or acoustic features [12], improved mechanical behavior (particularly elastic modulus) or better aesthetics. The products in which these materials can be applied range from sports equipment, furniture or bins.

Natural fibers and polymer composites for rotational molding have mainly been produced via dry-blending techniques [6,13,14,15,16]. These simple processes avoid the thermal degradation of the natural fibers during melt compounding [6,15] and also limit the size reduction from the extrusion process, which might not be desirable. However, compounding is also performed as it often achieves greater dispersion and adhesion between fiber and resin [6] and also allows for higher ratios of foreign materials use.

In RM fibers, the use of natural fibers has achieved increases in mechanical performance, particularly in elastic modulus, but often the impact performance is reduced [14,15]. Ortega et al. [15] reported increases of over 20% in the tensile modulus compared to the base PE resin. However, on this occasion, all other properties were inferior to the unfilled material, especially impact, which was reduced by over 90%. This study was undertaken with banana fibers and dry blended single-layer composites of up to 20%. The reduction in properties was associated with increased stiffness of the composite because of the high tensile modulus of the banana fibers.

Many studies in this field also adopt the method of producing dual or multilayer structures to improve performance. Multilayered structures are an easy method to reinforce, and they achieve specific performance for application and service environments, for example, using different thicknesses in each layer [13,17]. An example of multilayer structures, again with banana fibers, is found in the work undertaken by Ortega et al. [15]. In this work, different approaches were taken to achieve two- and three-layer structures at 5 wt% banana or abaca fiber. The presence of the banana fibers increases the tensile and flexural modulus by 188% and 200%, respectively. The three-layer structure also reduces the drop in impact strength; however, it was significantly affected, achieving just 68% of the virgin PE value [15].

The purpose of this study is to compare compounded banana fiber composite performance prepared from untreated fibers to allow an understanding of the different processing methods. As mentioned, Ortega et al. [15] have previously studied single-layer and multilayer rotationally dry-blended and multilayer composites. Cisneros Lopez et al. [18] assessed polylactic acid (PLA) and agave fiber composites produced via the dry-blending technique. A comparison of compression and rotational molding found a significant difference in porosity and therefore performance. The porosity of up to 80% in rotationally molded specimens resulted in reductions in performance with increased loading. Similarly, other studies from Suárez et al. [19] with giant reed fibers and PLA/PE composites reported a strong correlation between increased fiber loading, increased porosity, and thus reduced impact and tensile strength. The differences were attributed to the nature of the shear-free rotational molding process, poor compaction, and increased agglomerates. Other studies with PLA and PE dry-blended buckwheat husk fibers were undertaken by Andrezejewski et al. [18,20]. A significant reduction in properties was observed, due to the nature of the rotational molding process, and the fillers were thought to impede the polymeric particles from joining into a solid material. A consideration of effects on rheology found increases at all addition rates in complex viscosity, particularly in the low shear region. Anisko et al. [21] compared dry-blending and melt compounding of bio-based PE and expanded vermiculite composites. In this case, the melt compounding technique resulted in improved quality of composite parts. Dry-blended samples were reported to have a higher porosity and thus inferior impact strength and tensile properties; in some cases, for example, 10 wt% loading tensile strength was 400% higher for compounded composites. This study aims to collect and discuss comparative data using a different technique, i.e., melt compounding, which has yet to be assessed with banana fibers.

This rotationally molded composite has been prepared via single screw extrusion at 20 wt% banana fiber loading. Single, dual, and multilayer structures were manufactured given the most superior mechanical performance in many other studies. Therefore, the process time to obtain these multilayer structures was assessed together with the processability of the compound. The mechanical and thermomechanical behavior of rotomolded parts was assessed, also determining the influence of the pulverization process on the fiber size.

## 2. Materials and Methods

### 2.1. Materials

Revolve N-269 is a rotational molding metallocene a low-density metallocene hexene hexane polyethyelne grade (mLDPE) supplied by Matrix Polymers. The material has a nominal density of 0.940 g/cm^3^ and a melt flow index of (MFI) 4.0 g/10 min (190 °C/2.16 kg).

Banana fibers were extracted at Universidad de Las Palmas de Gran Canaria. They were extracted from the superimposed leaves that form the pseudostem. Fibers were used without any prior treatment in order to avoid any chemical residues and to keep the environmental footprint of the fibers as low as possible. Nevertheless, the fibers were shown to have a decomposition temperature (in an air atmosphere) of over 210 °C [15], which is lower than the one used for processing in both stages (melt compounding and rotomolding).

### 2.2. Methods

#### 2.2.1. Composite Preparation

The PE compound was prepared with the N-269 material and banana fibers. The composite was prepared by dry-blending 80% and 20% MDPE and banana fibers, respectively. The mixture was then compounded using a Collin single-screw extruder. Processing was performed at 120 rpm and output rate at 1.5 kg/h. The barrel conditions are presented below in Table 2. Temperatures were low relative to typical polyethylene processing to prevent and protect the natural fiber from thermo-degradation.

The extrudate was pulverized on an Orenda AF Lab Airforce mill/pulverizer, keeping temperatures between 30 and 40 °C. The pulverized materials were rotationally molded using a Ferry Carousel RS 1.9 biaxial manufacturing machine, using a hexagonal mold (300 mm × 260 mm, 1200 g shot weight) and the following processing conditions: oven temperature of 260 °C, 8.0 rpm major axis and 1.9 rpm on the minor axis. The internal air temperature (IAT) and peak internal air temperature (PIAT) were monitored using the 493 K K-Paq and the K-kord software (v3.0.0). The IAT at removal from the oven was 200 °C for all molded parts. The cycle time was recorded from 40 °C to a PIAT of 210–215 °C before demolding at 90 °C. The cycle time was evaluated on the K-kord software. Each molded part was cooled in forced air for 15 min before demolding. The different structures prepared are outlined in Table 3.

Specimens for the tensile and flexural analysis were cut from the rotationally molded parts using an ISO 527 [22] and ISO 178 [23] die on a pneumatic press for mechanical analysis, while impact plaques of around 125 mm × 125 mm, complying with the ARM Cold Impact standard, were also prepared.

#### 2.2.2. Fiber Size Analysis

Composites in the granule and powder were compression-molded. This pressing technique used 1 g of composite material and 1 g of natural material at 200 °C for 120 s. This was repeated on the sample to ensure the fibers were well dispersed and distinguishable individually.

Fiber size distribution was obtained by analyzing the sheets, which were scanned in a Canon Lide 400 Scanner at a high resolution. Images were then treated to remove background and by segmentation, obtaining a black-and-white figure. The free software ImageJ (v1.54e) was used to obtain the fiber-size distribution, using the parameter ‘feret diameter’ to obtain the fiber lengths, following the procedure described by Suárez et al. [24].

#### 2.2.3. Thermal Behavior—Differential Scanning Calorimetry

Mettler Toledo DSC823e was utilized to assess the melting temperature, the heat of fusion, and the crystallization temperature. Samples of 5–10 mg were used for these tests. Two heating and cooling cycles from 50 to 190 °C at 10 °C/min were used to remove the thermal history and collect data similar to the RM processing cycle.

Crystallinity was calculated based on the following equation [25]:

(1) $$\mathrm{Percentage}\;\mathrm{Crystallinity}\;\left(\mathrm{Xc}\right)=\frac{\Delta h}{\Delta h_{\mathrm{fusion}}\cdot w_{mLDPE}}\times 100$$

(2) $$\mathrm{Weight}\;\mathrm{Fraction}\;\mathrm{of}\;\mathrm{mLDPE}\;\left(w_{mLDPE}\right)=\frac{m_{mLDPE}}{m_{BFC}}=0.8$$

where Δh is the enthalpy of fusion of the polymer and respective composite and Δh_fusion_ is the specific enthalpy of fusion for PE, evaluated as 293 J/g (theoretical enthalpy of 100% crystallinity PE [26]).

#### 2.2.4. Rheological Assessment

Rheological behavior was assessed for all formulations in a TA Instruments HR10 Discovery Rheometer. The tests were carried out at 190 °C using 25 mm stainless steel parallel plates and a 1 mm geometry gap. The frequency sweep was undertaken at 1.0% strain, between an angular frequency of 100 and 0.1 rad/s.

#### 2.2.5. Density Measurement

The part density of each composite structure was assessed using the Mirage, Electronic Densitometer SD-200L (Oakland Instruments, Shakopee, MN, USA). Data were collected from rotationally molded articles, measuring a minimum of 5 samples.

#### 2.2.6. Mechanical Properties

Tensile tests were run according to the ISO 527 standard at a test speed of 5 mm/min. Specimens were tested at room temperature (23 °C) after 24 h conditioning at room temperature. The Lloyd X LRX instrument and Nexygen software (v3.0) were used to calculate the tensile strength, elongation at break, and tensile modulus. The Lloyds Instruments LRX machine was also used to undertake the ISO 178 standard. Data were collected for the flexural properties at a test speed of 5 mm/min. A preload of 2N was set for each sample before results were recorded.

ARM impact testing was undertaken using the dart drop system. Plaques were impacted at −40 °C, after 48 h at room temperature and 24 h conditioning at testing temperature, using a fixed weight of 4.54 kg (10lb) dart for all testing.

#### 2.2.7. Dynamic Mechanical Thermal Analysis

Dynamic mechanical thermal analysis (DMTA) was performed to assess the viscoelastic behavior of the composite structures. This technique was undertaken on the Tritec 2000 device from Triton Technology. The analyses were conducted from −170 °C to 90 °C at a heating rate of 2 °C/min, in dual cantilever flexural mode, and 5 μm displacement with a frequency of 1 Hz. Data across the temperature range for the Storage (E′) and Loss (E″) modulus were collected in addition to tan δ.

#### 2.2.8. Optical Microscopy

Microscopy was used to assess the cross-section of the and the interface between each layer of material. The Veho Discovery Microcapture VMS-004 captured images of the layered interface. The magnification selected for imaging was ×10.

## 3. Results and Discussion

### 3.1. Cycle Time Assessment

Dependent on the dual or multilayer structure, the cycle time was increased by 14.9–32.3%. The addition of each shot of material required an extra 4–8.5 min (two-layer to three-layer) compared to the monolayer mLDPE material. All cycle times are presented in Table 4. The cooling rate appeared to be unaffected by the presence of fibers in the material. Although one of the benefits of using fibers is the reduction in the amount of raw material (polymer) used, if multiple shots are required, the longer cycle times will increase gas consumption. However, as the industry moves toward electrically heating tooling and increased automation, this drawback might have a lower environmental and practical importance.

**Figure 2:**
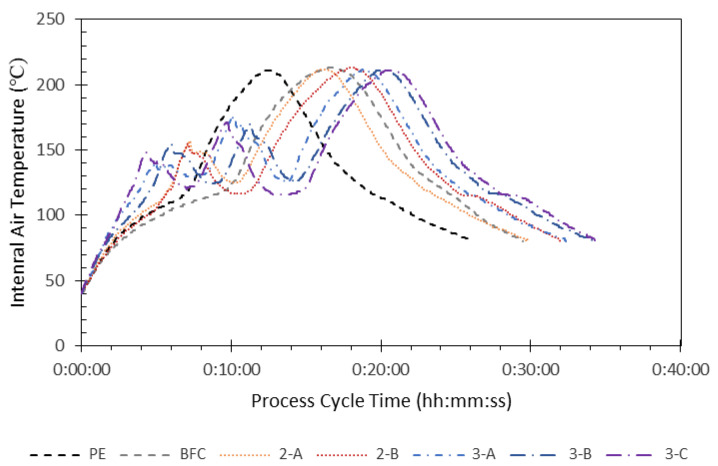
Internal air temperature and process cycle time curves from the processing of all materials for characterization.

The composite (BFC) single layer experienced an extended heating cycle compared to mLDPE (14.7% longer). This is expected to be due to the high complex viscosity in the low shear region and the insulation of the porous structure caused by the presence of fiber. The cooling cycle was on the contrary unaffected for the BFC compared to the mLDPE. Similarly, Ortega et al. [17] obtained an increase in the total cycle time for composites with banana fibers, with a reduction in cooling time; López-Bañuelos et al. [27] also obtained a reduction in the cooling cycle time when working with agave fibers, attributed to less insulative polymeric material present in the composite, resulting in less heat transfer resistance and therefore an accelerated cooling. The different cycle time for the dual and multilayer structures is due to the different amount of material used in each layer; apart from this, the same observations arise: the higher content in the compound (that is, in banana fibers) resulted in a delayed PIAT. Figure 2 presents the longer cycle time of BFC compared to neat PE, which is closer to the time needed for obtaining parts with different layers.

### 3.2. Fiber Characterization

**Figure 3:**
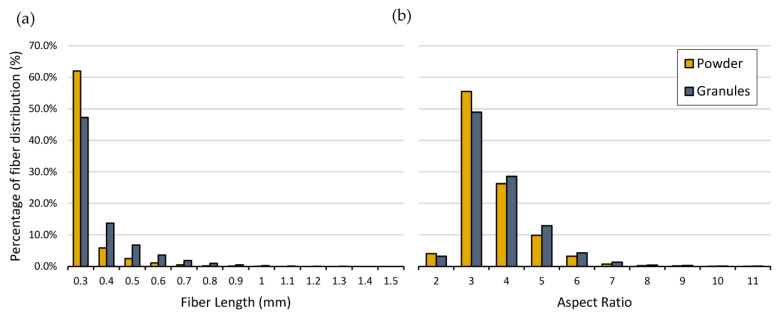
(**a**) Fiber length size distribution on granules (extrudate) and powder (pulverized material), (**b**) aspect ratio distribution for granule and powder.

**Figure 4:**
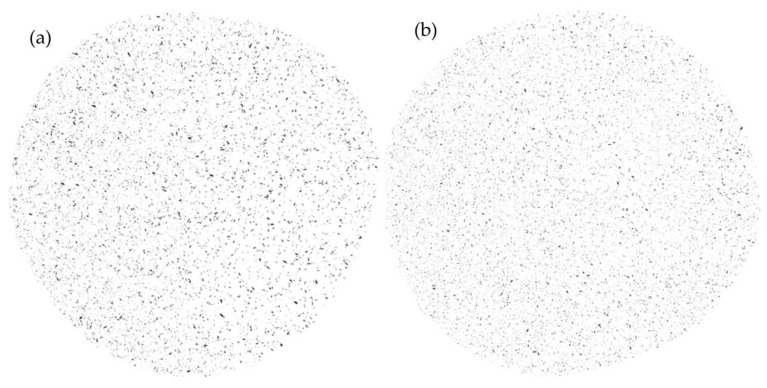
Powder and granule comparison of powder and granule composites (before and after the pulverization process). Hot platen-press of (**a**) granule and (**b**) powder.

Fiber size and aspect ratio were assessed before and after the pulverization process, that is, on the extruded granules and on the powder used for RM. Upon initial preparation, fibers were around 3 mm in length. The observations after extrusion found 47.2% and 13.7% existing at 0.3 mm and 0.4 mm, respectively, as shown in Figure 3a. The extrusion process resulted in a reduction to only 10% of the length originally blended with polyethylene, significantly reducing the length scale within the particle size distribution. The effects of pulverization were not as significant as those from fiber to composite; however, further reductions were recorded. A visual representation of the pressed sample is presented in Figure 4.

Pulverization increases the finer (<0.3 mm) content to 62%, an increase of over 30% compared to the extrudate. Finally, in the pulverized composite, 75% of the size distribution existed below 0.4 mm. This is expected to have an influence on the rheological properties as well as the mechanical performance of the composite.

The aspect ratio, defined as the ratio of the lateral dimension and the height of the fiber, was also affected due to the material grinding. The distribution of the aspect ratio recorded a shift to a smaller ratio distribution after the pulverization process (Figure 3b), which is expected due to the significant reduction in length. The fibers’ diameter is not affected by the pulverization, and so it does not compensate for the decrease in the aspect ratio (length reduction). Higher aspect ratios are expected to offer greater degree of transfer of stress to the polymer matrix; as a result, it contributes significantly to mechanical performance [28]. For example, Kwon et al. studies highlight that larger aspect ratios demonstrate a higher improvement than smaller aspect ratio fillers in hybrid composites [29]. As a result, it is likely that the pulverization process has reduced mechanical performance by reducing the aspect ratio; however, this was an unavoidable step to produce powder for RM.

### 3.3. DSC

The addition of banana fibers at 20 wt% by melt compounding had a significant effect on the thermal characteristics, as shown in Table 5. Before the size reduction achieved from pulverization, fiber introduction resulted in an increase in melt temperature by almost 8 °C, in addition to a 14% reduction in the overall crystallinity, compared to mLDPE. The presence of the fibers possibly disrupted the polyethylene crystal configurations, thus reducing the overall crystallinity. The pulverized material, proven to have a smaller fiber size distribution, exhibited a 10% reduction in crystallinity. Bouafif et al. [30] observed changes in the crystal conversion and increases in crystallinity with wood particle sizes of 0.15 mm–0.35 mm, similar to those in the BFCs. This was attributed to the wood fibers having the ability to heterogeneously nucleate the HDPE. This was not observed within the BFC, as also shown in other research works using lignocellulosic fillers in rotational molding. Another study with PLA and PE, by Suárez et al. [19], observed no difference with the addition of reed fibers; in this case, fibers were 3–4 mm in length and up to 10 wt%. Crystallinity was reduced with the addition of fibers due to a reduction in the melting enthalpy. Further researchers reported minimal changes in thermal transitions for polyethylene due to the rapid nature of the crystallization process for this material [17,19,20].

### 3.4. Rheology

The presence of the fibers increased the viscosity in the low-shear region (0.1 rad/s) in the extrudate and the powder. The low shear region was pointed out by Martin et al. [31] as a significant factor related to the sintering stages of the rotational molding process. Generally, the lower the viscosity, the better the moldability, and faster sintering can occur due to greater relaxation between particles. Hence, this is the region of interest for this study.

**Figure 5:**
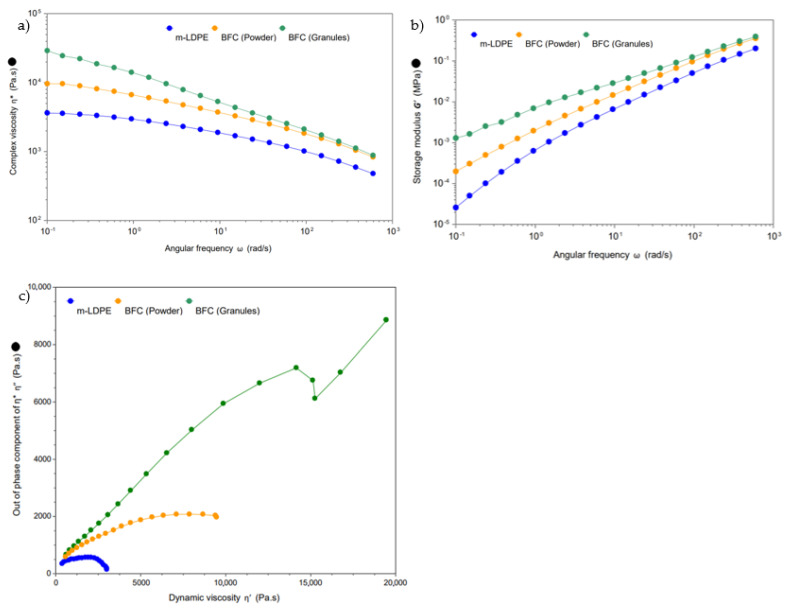
(**a**) Complex viscosity vs. angular frequency for frequency sweep tests at 190 °C for the virgin PE, composite granule, and composite powder. (**b**) Storage modulus (G′) vs. angular frequency, frequency sweep at 190 °C. (**c**) Cole–Cole plot for neat PE and composite materials in both powder and granule forms.

As presented in Figure 5a and Table 6, the incorporation of fibers increases the viscosity, as is otherwise expected. It is also observed that the extrudate increases the mLDPE viscosity by a factor of 7, while the pulverization process reduces this significantly. The pulverization process resulted in a value only 2.7 times greater than the mLDPE, which is explained by the reduced length of the fibers due to the grinding process. The larger particles have more fiber–fiber interactions and form a 3D network [32], which is less prevalent with smaller particle size. Rueda et al. [33] observed this phenomenon with short and long glass fibers, related to the higher aspect ratio of larger fibers.

Higher angular frequency presents a similar course for G′ and η^*^ for both powder and granule BFC, which is attributed to the orientation of the fibers occurring that allow strain softening and less agglomeration of the fibers [33]. However, in both cases, the presence of the fibers in the composite increases the overall viscosity. Approaching lower angular frequency results in different flow regimes as BFC granule appears to be more dependent on the frequency.

Increases in storage modulus with the addition of the banana fiber were found (Figure 5b), showing an increase in the elasticity of the composite melt [32]. Generally, the greater the increase, the stronger the network, highlighting again that there are greater interactions and greater networks with the higher aspect ratio of BFC granules rather than powder.

It is also possible that fibers could be attracted to one another and flocculate in the matrix; this, accompanied by greater particle size, results in higher complex viscosity [28]. Greater compatibilization with agents like PE-g-MAH or fiber pretreatment may improve this, while the beneficial effects of such additional materials on rotomolding are still unclear.

The loss modulus is higher than the storage modulus for most of the studied range, which demonstrated the predominant viscous behavior of the melt. Table 6 shows the cross-over point (G′ = G″), which indicates the transition from viscous to elastic behavior [34]. In this case, the cross-over occurs at a lower angular frequency for the BFC compared to the mLDPE, which is related to a more solid-like behavior of the composite [34]. Köpplmayr et al. [35] outlined that the cross-over point also describes the degree of homogeneity in the composite, whereby a lower frequency suggests finer dispersion and higher frequencies suggest phase separation and agglomeration. As a result of the powdering process and the particle size in such composites, it seems that there has been an increased agglomeration compared to the granule BFC. In addition, the BFC (powder and granule) G′ and G″ were increased compared to mLDPE, showing the reinforcement provided by the lignocellulosic fibers.

The Cole–Cole plot in Figure 5c shows the imaginary (η″) plotted against the dynamic/real viscosity (η′). The presence of the start of a second arc for the BFC granule and distortion highlights a complex structure with two phases and some separation between the fiber and the matrix. The same phenomena were reported by Ou and Song [36], who attributed this behavior to deficient compatibility between the wood filler and the polypropylene. In that case, a reduction in agglomeration and phase separation was achieved using lubricants. The use of compatibilization agents may improve the adhesion between fiber and matrix, thus resulting in a curve closer to a semicircle. As also observed for the complex viscosity, the most significant change was recorded with the larger fiber size from BFC granules, while the behavior of the ground pellets did not deviate greatly from the optimal semicircle shape.

### 3.5. Composite Part Density

The addition of fibers via melt compounding resulted in a lower density compared to virgin PE. The BFC exhibited a density of 0.6942 g/cm^3^, while for the neat mLDPE, it was 0.9380 g/cm^3^; the reduction in density can be attributed to an increase in porosity of the material, as reported by other studies [18,20]. 

**Figure 6:**
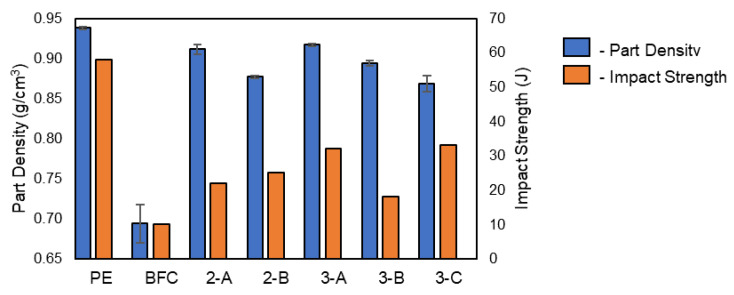
Part density of rotationally molded articles.

Figure 6 shows the density and impact strength plotted for each composite structure. For the virgin PE and BFC there is a clear relationship between changes in density and impact strength; this is also the case for the elongation at break, the tensile strength, and Young’s modulus. The layered structures, due to a higher proportion of virgin material, recorded a lesser reduction in density, while, at the same time, impact performance and tensile and flexural properties were greater than for BFC. The density was higher for the symmetrical structure with the same shot weight of virgin on the internal and external surface.

### 3.6. Mechanical Analysis

**Figure 7:**
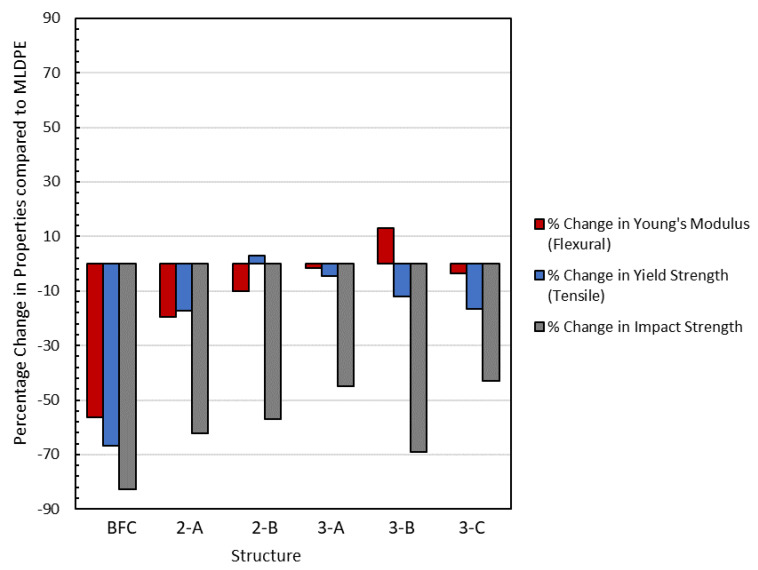
Mechanical performance as a percentage change compared to neat polyethylene.

The mechanical results are summarized in Table 7, and statistical analysis is provided as Supplementary Material (Table S1). The percentage change from the neat polymer material, with the same total weight, is presented in Figure 7. Generally, the properties are reduced by up to 68%, with the most significant decrease recorded in impact strength. A reduction in this property was expected within this range, as reported in other studies. For example, Anisko et al. [21] reported a reduction of over 50% at a 10 wt% addition of vermiculite [21]. However, this contrasts with studies from Jayaraman et al. [37], where an increase of 17.5% was reported with the addition of sisal and wood fiber composites prepared via melt compounding. The reduction in impact strength was attributed to a porous cross-section offering many sites for propagation. The lack of coverage on some internal layers with the natural fibers would also not improve this performance. However, when the shot weight of the internal layer was increased, the reduction in impact strength lessened by up to 20%. This provides a possible solution to overcome the impact strength reduction when using foreign materials. In all cases, elongation was significantly compromised as the elastic performance of the polyethylene was reduced with the addition of the rigid fibers.

As seen from the statistics, BFC exhibits significantly lowered flexural modulus and tensile strength, due to the high porosity of these samples, which reduce the transmission of the mechanical loadings. However, the tensile modulus is not significantly affected due to the BFC incorporation; the high stiffness of the fibers might be counteracting the reduction in properties to the voids in the sample. Sample 2B (450 + 300/450) presented a slight (not significant) increase of 10% in Young’s tensile modulus; other studies reported improvements of much greater magnitude with dry blended banana fibers [15]. However, such results were obtained with fibers at longer aspect ratios. For samples made in two layers, it is interesting to highlight that 2-A exhibits significantly reduced mechanical properties compared to the neat PE, possibly due to the presence of voids resulting from the fiber incorporation. This effect is counteracted in the structure 2-B, with a similar value of density, but with lower fiber content in the layer (20% for 2-A vs. 8% for 2-B); therefore, it seems that fibers may act as an active reinforcement of the matrix but at lower loadings. An excessive amount of foreign materials results in high values of porosity, poor distribution, fiber entanglement and, thus, a worsening of mechanical properties. In fact, only the elongation at break and impact strength are significantly modified for 2-B compared to neat PE (Table S1), thus leading to the conclusion that the incorporation of banana fibers in this two-layer structure, at a 5% overall fiber content, is not affecting the mechanical properties of the final part, while potentially increasing its sustainable character. The incorporation of wheat bran into a PE matrix also shows a dependance between the amount of filler and the mechanical properties [38]. Other authors have obtained even higher drops in mechanical properties for low ratios of lignocellulosic fibers, such as hemp fibers (50% reduction of tensile strength for 5% composites) [16]. In general terms, it is accepted that the use of fibers with a long aspect ratio and high rigidity [39] can improve the elastic modulus of the composite, while the tensile strength can be compromised, especially at loadings over 5% [19]. The use of chemical treatments, such as the pre-impregnation of fibers with maleic anhydride polyethylene, could prevent this reduction in tensile stress, particularly if high loadings are intended [39].

Finally, the effect of the composite location in the thickness has also been demonstrated; only formulation 3-A, which has the composite at the center of the part, provides similar properties to the neat matrix, except, as already analyzed, for the impact strength. The modification of the composite by adding inequal shots in the internal and external layers results in a reduction in mechanical properties.

Tensile modulus values recorded for the three-layer structures remained within 10% of the polyethylene resin. Increases of 10% were achieved for 3-B but much less than those reported by Ortega et al. [15] of up to 188%. Again, it is important to consider that that work was performed with fibers with a long aspect ratio, prepared via dry-blending, and thus not suffering from size reduction during the preparation, while also being more limited in the ratio of fibers incorporated.

The flexural modulus improved by almost 13% for 3-B (600/300/300), as achieved by the multilayer structure. The presence of the fibers, with a higher modulus than polyethylene, can be responsible for this improvement, accompanied by the greater external thickness of PE where the loading was applied. Ortega et al. [15] adopted a dry-blending technique where fibers were much greater in length and would have experienced less reduction during processing, thus offering a greater extent of reinforcement. Finally, that work was able to only include 5% fiber in the shots, meaning a maximum of less than 4% of fiber in the overall part. The results providing the best results in that reference were obtained in a two-layer structure, with an overall amount of 1.25% in the part. The use of a previously compounded material in the work presented here has allowed the ratio of fibers introduced to increase, without significantly reducing the mechanical performance of the composites, apart from impact strength, which was even more pronounced in the mentioned work.

When comparing structures 2-B and 3-A, with the same internal layer, and the same overall amount of fiber, no significant differences are obtained. The difference between these two parts is the molding in two (2-B) or in three (3-A) shots but using the same distribution of materials. The lack of differences between their behavior indicates that the two-layer constructions are more favorable due to the lower cycle time and the easier molding.

### 3.7. Composite Molded Articles

**Figure 8:**
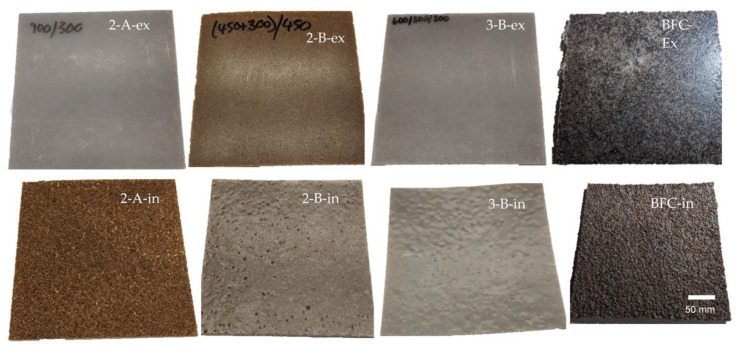
Images of rotationally molded multilayered composites. Samples are individually labeled, where ‘ex’ and ‘in’ correspond to the external and internal surfaces, respectively (sample size 300 mm × 260 mm).

The multilayer composites showed good dispersion of fibers throughout all assays. The coverage of the internal polyethylene layer was dependent on the shot weight. At lower shot weights and therefore less volume of material, pitting was observed on the internal surface. The fibers were thought to impede the flow of the powder and the polymer melt due to the random orientation protruding from the surface. Even when complete coverage is achieved, at higher shot weights, an uneven surface is present, perhaps as a result of the fiber positioning. The monolayer banana fiber composite appeared significantly darker than the multilayer structures, as shown in Figure 8. This is potentially due to the extended exposure to the rich oxygen environment inducing thermo-degradation of the fibers during processing, as well as to the longer time that the fibers were subjected to the temperature.

Multilayer structures did not present this effect, potentially due to the polyethylene layers offering some insulative protection to the fiber.

**Figure 9:**
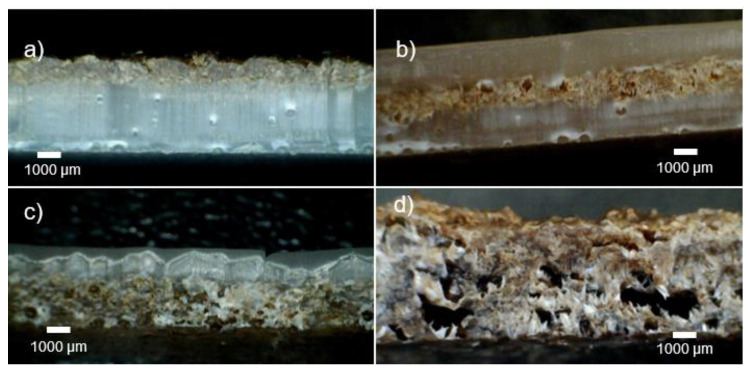
Cross-sectional images of composite layered structures (**a**) 2-A, (**b**) 3-A, (**c**) 2-B, and (**d**) BFC.

Some porosity and voids were present in the composite layer, resulting from the release of moisture of the lignocellulose fibers and the significant increases in the viscosity in the low-shear process environment. Cross-sections of the molded articles are presented in Figure 9, where the porosity shown in Figure 6 is evident in the BFC and multilayered composites.

### 3.8. Dynamic Mechanical Analysis

**Figure 10:**
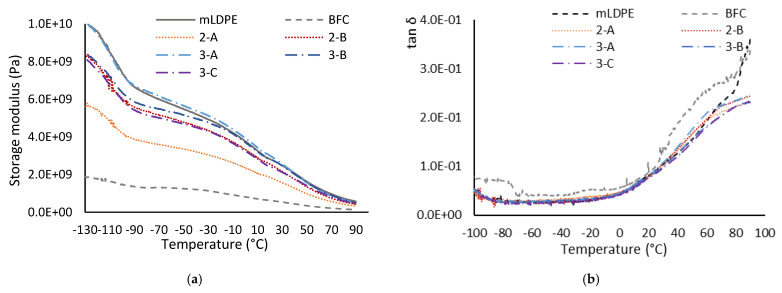
Results from the DMA analysis for the rotomolded samples: (**a**) storage modulus, (**b**) damping factor.

The analysis of storage modulus (E′), loss modulus (E″), and damping factor (tanδ) with temperature allows for the evaluation of the changes in the mechanical properties due to the addition of fibers and shredded material to the HDPE matrix (Figure 10). DMA is an essential test for the evaluation of the mechanical properties of materials due to its sensitivity to structural changes, including the interfacial bond between the fibers or fillers and the matrix [40].

The DMA storage moduli (Figure 10a) are indicative of the elastic behavior of the materials. All composites exhibit a lower storage modulus than neat mLDPE, meaning that the lignocellulosic material does not create a stiffening effect in the temperature range studied; the difference is particularly relevant for under-zero temperatures, whereas the elastic behavior of the different parts tends to converge from room temperature. The BFC samples show the lowest elastic behavior in the entire range, even at high temperatures; this behavior was also observed for mechanical testing, where this series exhibited a significantly reduced mechanical performance. This reduction in properties reflects that the amount of fiber used (20%) is excessive for rotomolded parts. It is also observed that the manufacturing in layers results in better mechanical behavior, as also found in tensile and flexural testing. Comparing the behavior of parts made in two layers (2-A and 2-B), which have the same amount of fiber but distributed differently in the part thickness, the beneficial effect of reducing the amount of fiber in the external shot is clear; part 2-B, with an external layer made of compound plus virgin material and an internal one made only of banana composite, exhibits a behavior closer to the neat PE than part 2-A, made of an external layer of composite and internal one of neat mLDPE. If the multilayer (three-shots) parts are analyzed together, it can be seen than the part made with the banana fiber in the core of the part exhibits the same behavior than the PE. This would suggest that layering the composite between equal amounts of PE is more beneficial than distributing these outer and inner layers in different thicknesses. However, as also observed for the two-layer constructions, the use of a thicker outer layer seems to yield better behavior than the opposite; that is, the use of neat PE is required in the external layer to provide acceptable mechanical properties in multilayer structures. Finally, it is observed that the storage modulus decreases in an expected constant way [20]. Apart from the different values for E′, the trend of the curves is the same, regardless of the ratio of fiber or the number of layers. The incorporation of lignocellulose fibers into PE matrices usually leads to an increase in storage modulus, at least for injection-molded samples [40], while the trend is different for rotomolded samples. Andrzejewski et al. [20] have reported no significant differences in the course of storage modulus for samples rotomolded with buckwheat husk, while this analysis was only made starting at 30 °C. In any case, the storage modulus for PE was found to be higher than for the composites, tending to arrive at similar values as the temperature increased, as also obtained in this research with banana fibers. The reduced stiffness of the composite might be due to the increased porosity, as also discussed above in this manuscript. Hejna et al. [38] reported no increases on storage modulus for wheat bran/LLDPE rotomolded composites in the −100 to 100 °C range, but also significant decreases. Similar conclusions arise from the previous analysis of composites obtained with banana fiber at lower ratios of fiber and obtained without the compounding stage [17]. In that case, the behavior is even more dissimilar to that of the neat PE matrix, despite the low amount (10% maximum) of fibers used, which suggests the beneficial effect of the compounding stage.

For the loss modulus (E″), the maximum PE value is found at about −110 °C and 42 °C, the first related to glass transition and the second one to the α-transition [38]. These transitions can also be observed in the damping factor plot (tan δ). These transitions remain almost unchanged for the composites, which is related to the lack of an effect of the fibers on the material stiffness and thermal behavior, as also observed from DSC analysis. Finally, no significant differences were observed from the tan δ plot, which might be due to the poor compatibility between the fibers and the matrix, despite the preparation stage of the compound by melt mixing.

From the DMA results, the adhesion factor (A) can be obtained, as described by Kubat et al. [41], who consider that the behavior of the composite is due to the properties of the matrix, the filler, and the interphase (Equation (3)):

(3) $$A=\frac{1}{1-x_{F}}\cdot \frac{tan\delta_{C}}{tan\delta_{PE}}-1$$

where $x_{F}$ is the ratio of the filler in the composite (in volume), and tan $\delta_{C}$ and tan $\delta_{PE}$ are the damping factors of the composite and the neat PE, respectively.

**Figure 11:**
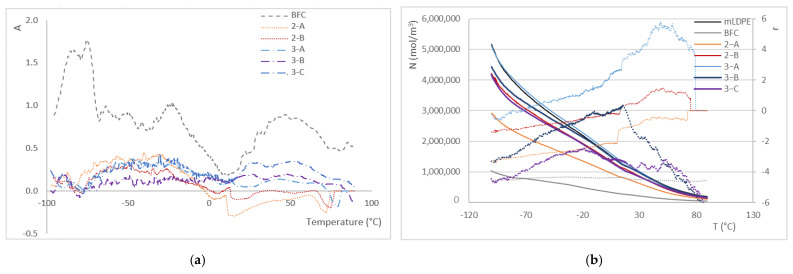
(**a**) Adhesion factor (*A*) and (**b**) entanglement factor (*N*, left axis; continuous line) and reinforcement efficiency (*r*, right axis; dotted line).

Lower values of A indicate higher interfacial adhesion, which should correlate with a higher mechanical behavior [38]. On the contrary, an increase in A is associated with higher damping and, therefore, with a higher energy dissipation due to chain movements inside the composite, insufficient interfacial adhesion, or higher porosity [38]. As this factor depends on the damping factor, it also varies with temperature (Figure 11a).

It can be seen that, as expected, BFC exhibits higher values of the adhesion factor, therefore demonstrating the lowest adherence between the banana fiber and the PE matrix. This is also a reflection of the high porosity of the part. On the other hand, the behavior of the remaining series regarding this parameter is closer to zero and shows less variability in the temperature range studied, which also indicates better thermal stability of the material. It should be clarified that the negative values of the adhesion factor obtained do not have any physical sense, and these are due to the simplification of the calculations and the removal of the interphase region [38,42].

Finally, different authors propose using the entanglement factor as an approximation of the intensity of the interaction between matrix and filler/reinforcement, together with the efficiency factor [43,44]. The entanglement factor is usually calculated at several temperatures to evaluate the potential differences in such interactions due to different service conditions; in this manuscript, the variation of this factor with temperature is shown in Figure 11b. The entanglement factor (N) is calculated using Equation (4):

(4) $$N=\frac{E^{\prime }}{RT}$$

where $E^{\prime }$ is the storage modulus at a certain temperature (T, in K) and R is the universal gas constant.

The reinforcement efficiency (r), calculated as shown in Equation (5), provides information about the filler/matrix interaction:

(5) $$E_{c}^{\prime }=E_{m}^{\prime }\cdot \left(1+r\cdot V_{f}\right)$$

where $E_{c}^{\prime }$ and $E_{m}^{\prime }$ are the storage modulus of the composite and the matrix, respectively, and V_f_ the percentage of the filler (by volume).

Again, the lowest values for N are obtained for BFC, in the whole temperature range, while 3-A shows the highest values, close to and even slightly higher than for neat PE, thus explaining the increase in tensile properties found in static tests at room temperature. The reinforcing efficiency of the fibers is under 0 until room temperature is reached, which means that the fibers do not act as a reinforcement until then. Furthermore, BFC, 2-A, 3-B and 3-C show a negative value along the entire range of temperature, related to the decrease in mechanical properties already described above. So only in the series 2-B and 3-A can the fiber be considered as reinforcing the PE matrix, and above 25 °C, it is more significant for the three-layer parts. After about 60 °C, the reinforcement efficiency tends to decrease again, although for 3-A, the reinforcement character is still significant.

## 4. Conclusions

This study characterized and evaluated the thermal, rheological, and mechanical properties of mLDPE banana fiber composites. An analysis was undertaken on rotationally molded articles and in single-layer and multilayer structures, where consideration was also given to processing cycle time.

The preparation required to produce the rotational molding powder demonstrated a reduction in the fiber length and aspect ratio, thus influencing the rheological properties. A 7- and 2.7-fold increase was observed for viscosity after extrusion and pulverization, respectively, compared to the based polymer. Size reduction showed lower complex viscosity in the lower shear region due to a reduction in fiber–fiber collisions and restriction of flow due to the degree of percolation.

Increases in cycle time were observed in all cases using the composite, in single-, dual-, and multiple-layer structures. The cycle time was increased in up to 32.3% in total for the three-layered structures.

The impact strength of all composites was significantly reduced, demonstrating once again the high sensitivity of rotomolded parts to the incorporation of foreign materials. However, slight improvements in elastic modulus were found for some of the composites. Considering the properties of the composites and the cycle time, the manufacturing in two layers with an external shot containing the fibers seems to be the optimal manufacturing strategy. The melt compounding of the fibers allows one to obtain a relatively high loading for the incorporation of up to 8% of fibers in a shot, resulting in a 5% overall fiber content, without significant reductions in the performance of the molded parts.

Improvements of up to 13% were observed in Young’s modulus for structure 3-B, while other structures like 2-B and 3-A offered a similar tensile modulus (within 10% of virgin PE). DMA results confirm the observations made from static mechanical testing, finding significant worsening in the elastic behavior of parts made completely of the composite and demonstrating the potential of working in layers to improve the mechanical performance of rotomolded composites. It should be highlighted that composites with up to 15% fiber have been produced (total amount of fiber in the part) in two-layer structures, where the importance of using neat PE in the external layer has been highlighted. For three-layer structures, the part made with the composite as the core of equal PE layers in both sides provides a behavior closer to the neat matrix, while the increase in the thickness of the outer layer results in improved flexural behavior, as also demonstrated by the reinforcing efficiency and adhesion and entanglement factors.

## Figures and Tables

**Table 1:** Benefits and sustainability considerations for the use of natural fibers within polymer composites [9,10,11].

Benefits of Natural Fibers	Considerations to Ensure Sustainability
Derived from natural products	Local source, reduction in transportation emissions. Preferably coming from wastes or by-products
Further use of organic materials funds sustainable farming and generates greater value of products	Full life cycle not always considered, for example, the supply chain
Reduction in CO_2_ due to carbon capture, reduction in emissions compared to synthetic fibers (around 82–90% less)	Difficulty in feedstock traceability can leave questions about sustainability.
‘Making something from nothing’	Socioeconomic, ethical consideration

**Table 2:** Single screw extrusion conditions for the production of banana fiber composite.

Zone	1	2	3	Nozzle
Temperature (°C)	82	113	119	114

**Table 3:** Structures of rotationally molded articles obtained from single, dual, and multi-shot processing.

Structure	Structure Layers	Mass (g)	Formulation Name
Monolayer	mLDPE	1200	mLDPE
	BFC (Banana Fiber Composite)	1200	BFC
Dual Layer	mLDPE − BFC	900 − 300	2-A
	(mLDPE + BFC) − mLDPE	(450 + 300) − 450	2-B
Multi-layer	mLDPE − BFC − mLDPE	450 − 300 − 450	3-A
	mLDPE − BFC − mLDPE	600 − 300 − 300	3-B
	mLDPE − BFC − mLDPE	300 − 300 − 600	3-C

**Table 4:** Cycle times from rotational molding processing of banana fiber composites in mono-, dual-, and multilayer composite structures.

Material	Heating Cycle (mm:ss)	Cooling Cycle (mm:ss)	Overall Cycle (mm:ss)	Rate of Cooling (°C/min)	Increase in Cycle Time (%)
mLDPE	12:48	13:12	26:00	9.92	-
BFC	17:04	12:46	29:50	10.1	14.7
2-A	16:28	13:25	29:53	9.84	14.9
2-B	18:13	13:46	31:59	9.66	23.0
3-A	18:48	13:34	32:22	9.73	24.5
3-B	20:12	14:01	34:13	9.42	31.6
3-C	21:05	13:19	34:24	9.83	32.3

**Table 5:** Thermal Properties of mLDPE and BFC from DSC analysis.

Material	T_m_ (°C)	T_c_ (°C)	Δh (w/g)	X_c_ (%)
mLDPE	120.09	114.90	114.68	39.10
BFC-granule	127.82	110.77	75.37	32.15
BFC-powder	124.10	113.24	87.25	37.23

**Table 6:** Rheological properties of the mLDPE and composite in the low-shear region, which simulates the rotational molding environment.

Material	Complex Viscosity at 0.01 rad/s	Cross-Over Point (G′ = G″)
	(Pa·s)	Frequency (rad/s)
mLDPE	3600	>600.000
BFC-granules	25,800	399.605
BFC-powder	9700	543.603

**Table 7:** Tensile and flexural impact performance assessed on each rotational molded article (E: modulus, σ: strength).

Specimen	Flexural E (MPa)	Flexural Force (N) (at 5 mm Deformation)	Tensile σ (MPa)	Tensile E (MPa)	Elongation at Break (%)	Impact Strength (J)
mLDPE	548.7 ± 60.2	25.3 ± 1.1	17.5 ± 0.2	167.3 ± 0.7	268.2 ± 63.8	58
BFC	238.7 ± 5.9	28.5 ± 1.2	5.8 ± 1.0	144.9 ± 38.5	6.3 ± 6.3	10
2-A	440.6 ± 54.3	24.0 ± 0.9	14.5 + 0.5	111.9 ± 10.3	59.4 ± 8.6	22
2-B	493.3 ± 24.3	29.8 ± 1.8	18.0 ± 2.3	147.6 ± 7.6	21.5 ± 3.2	25
3-A	540.3 ± 28.5	25.2 ± 1.5	16.7 ± 0.2	185.1 ± 7.4	44.4 ± 6.9	32
3-B	620.9 ± 99.2	29.2 ± 2.1	15.4 ± 0.6	153.2 ± 17.4	53.3 ± 10.8	18
3-C	528.4 ± 41.4	33.0 ± 2.8	14.6 ± 0.6	153.2 ± 11.4	43.6 ± 7.2	33

## Data Availability

The datasets generated or analyzed during the current study are available from the corresponding author upon reasonable request.

## Supplementary Materials

The following supporting information can be downloaded at https://www.mdpi.com/article/10.3390/ma16206749/s1. Table S1: Statistical results (*p*-values) for the comparison of the flexural modulus of the different series (*p*-value < 0.05 reveals significant differences; *p*-value < 0.01 means very significant differences). Comparisons made on estimated marginal means (ANOVA analysis).
