# Supplementary material for: Mechanical Performance of Rotationally Molded Multilayer mLDPE/Banana-Fiber Composites
Source: Materials (Basel). 2023 Oct 18;16(20):6749. doi: 10.3390/ma16206749 (PMC10608177; doi:10.3390/ma16206749)
Supplement: Supplementary file 1 [file materials-16-06749-s001.zip › materials-2634521-supplementary.pdf]

Table S1. Statistical results (p-values) for the comparison of the flexural modulus of the different series (p-value < 0.05 reveals significant differences; p-value < 0.01 means very significant differences). Comparisons made on estimated marginal means (ANOVA analysis)

Material		Flexural modulus	Flexural strength (5 mm deformation)	Flexural strength	Tensile strength	Tensile modulus	Elongation at break
mLDPE	2-A	0.044	0.897		< .001	< .001	< .001
	2-B	0.650	0.006		0.984	0.510	< .001
	3-A	1.000	1.000		0.796	0.791	< .001
	3-B	0.350	0.024		0.021	0.860	< .001
	3-C	0.996	< .001		< .001	0.861	< .001
	BFC	< .001	0.091		< .001	0.433	< .001
2-A	2-B	0.699	< .001		< .001	0.063	0.737
	3-A	0.077	0.916		0.030	< .001	0.997
	3-B	< .001	0.001		0.715	0.015	1.000
	3-C	0.158	< .001		1.000	0.015	0.998
	BFC	< .001	0.006		< .001	0.083	0.377
2-B	3-A	0.795	0.005		0.358	0.050	0.975
	3-B	0.011	0.997		0.003	0.996	0.861
	3-C	0.938	0.081		< .001	0.996	0.987
	BFC	< .001	0.904		< .001	1.000	0.996
3-A	3-B	0.233	0.021		0.465	0.164	1.000
	3-C	1.000	< .001		0.040	0.164	1.000
	BFC	< .001	0.081		< .001	0.038	0.783
3-B	3-C	0.120	0.023		0.794	1.000	1.000
	BFC	< .001	0.996		< .001	0.989	0.518
3-C	BFC	< .001	0.005		< .001	0.988	0.853
